# Persistent postpartum depressive symptoms and developmental impairment in Bangladeshi children: a cohort study

**DOI:** 10.7189/jogh.16.04207

**Published:** 2026-07-03

**Authors:** Rasheda Khanam, Verna Mauren Amy Lazar, Nabidul Haque Chowdhury, Salahuddin Ahmed, Fahmida Tofail, Radiah Azmyne Khan, Pamela J Surkan, Diwakar Mohan, Sachiyo Yoshida, Sunil Sazawal, Fyezah Zehan, Abdullah H Baqui

**Affiliations:** 1 Department of International Health, Johns Hopkins Bloomberg School of Public Health, Baltimore, Maryland, USA; 2 Projahnmo Research Foundation, Banani, Dhaka, Bangladesh; 3 International Center for Diarrhoeal Disease Research, Bangladesh, Mohakhali, Dhaka, Bangladesh; 4 Department for Maternal, Child, Adolescents and Ageing Health, World; Health Organization, Geneva, Switzerland; 5 Center for Public Health Kinetics, New Delhi, India; 6 Department of Pediatrics and Child Health, The Aga Khan University, Karachi, Pakistan

## Abstract

**Background:**

Optimal child development across cognitive, language, and motor domains is essential for long-term health and well-being. Postpartum depression has been linked to adverse child developmental outcomes, but evidence on the impact of persistent postpartum depressive symptoms (PPDS) across these domains and over time remains limited, particularly in low- and middle-income countries.

**Methods:**

We retrieved data from the Alliance for Maternal and Newborn Health Improvement cohort, collected from 2017 to 2018. Our analysis included 799 mother-child pairs from Sylhet, Bangladesh. We assessed maternal depressive symptoms at 42–60 days and 12 months postpartum using the Patient Health Questionnaire – 9, and child development outcomes at 24 months using the Bayley Scales of Infant and Toddler Development, Third Edition. We used multivariable linear regression models to examine associations between PPDS (depression at both visits) and developmental outcomes in the cognitive, language, and motor domains in children.

**Results:**

Adjusted regression analyses showed that, compared to women with no depression, women with PPDS had significantly lower Bayle’s standardised scores in cognitive (−2.06; 95% confidence interval (CI) = −3.60, −0.52) and language domains (−3.13; 95% CI = −5.01, −1.25). We found no statistically significant difference in motor domain scores.

**Conclusion:**

We found that PPDS were associated with lower developmental scores in cognitive and language domains among children at 24 months in rural Bangladesh.

Child development involves the advancement of competencies in cognitive, social, emotional, motor, and language domains, among others [1]. These domains are cornerstones of human health and well-being, yet millions globally fail to reach their full developmental potential, particularly in low- and middle-income countries (LMICs). It is estimated that over 250 million children under five years of age in LMICs are at risk of suboptimal development due to poverty, malnutrition, and inadequate stimulation in early life [2].

The first 1000 days of life are a critical period for growth and neurodevelopment, during which early deficits in cognitive, language, motor, and socioemotional development may arise [3]. However, they also represent a key window of opportunity for interventions [3]. Prior research has shown that disruptions in early childhood development can have lasting implications on educational achievements, social functioning, and overall health later in life [4]. This burden is particularly pronounced in countries like Bangladesh, where high rates of stunting, poor caregiving practices, and limited access to early childhood development interventions compromise developmental outcomes [5]. A 2019 study on early childhood development revealed that 25% of children in Bangladesh were not meeting expected developmental milestones [5].

Numerous factors contribute to inadequate developmental potential, such as poverty, malnutrition, limited healthcare access, and family dynamics [2]. Understanding them is, therefore, important for reducing the burden and consequences of undetected developmental problems later in life. Maternal mental health, particularly depression in the postpartum period, is one such factor that has been shown to affect child development [2]. The World Health Organization (WHO) estimates that close to 20% of women in LMICs experience mental health disorders (mainly depressive disorders) after childbirth [6], which can have serious and lasting effects on both maternal and infant health. Untreated maternal postpartum depression has been associated with poorer cognitive functioning and adverse emotional and developmental outcomes in children, including increased behavioral problems and difficulties in establishing social relationships [7]. Maternal depressive symptoms have also been linked to impaired language acquisition and poor motor skill development in their children’s early youth [8]. These delays appear to be more intense in LMICs, where other socioeconomic stressors and limited access to mental health services compound the effects of depression [9].

The literature has documented the negative impact of maternal postpartum depression on child developmental outcomes [10]. However, only a few studies have investigated the effect of persistent postpartum depressive symptoms (PPDS) specifically on child development. In Brazil, Quevedo *et al*. [11] reported substantially lower language developmental scores in mothers with PPDS until 12 months postpartum, compared to mothers without depression at any assessment. While most studies have been conducted in high-income countries [12], emerging evidence from LMICs also shows similar findings [11]. Research from rural Pakistan found a significant negative association of chronic maternal depressive symptoms with child socioemotional development at 24 months of age [13]. This growing body of research highlights a need for a better understanding of how PPDS affects child development, specifically in LMICs. Here, we examine the impact of PPDS on child development across cognitive, language, and motor domains in a cohort of Bangladeshi children.

## METHODS

We retrieved data from the Alliance for Maternal and Newborn Health Improvement (AMANHI) [14,15], a well-characterised population-based pregnancy cohort of 3000 pregnant women recruited from a rural area in Sylhet district, Bangladesh, between August 2014 and December 2018. Women were enrolled after providing informed consent and following ultrasound confirmation of their pregnancy being at <20 weeks of gestation. They were followed up through their pregnancies and to two years postpartum, with data collected on their health and their children’s health.

### Data collection

In the AMANHI cohort, locally recruited and trained community health workers (CHWs) with a minimum of 10th-grade education made two-monthly home visits to identify pregnancies. The CHWs conducted four structured home visits during pregnancy: before 19 weeks (baseline), at 24–28 weeks, 32–36 weeks, and 38–40 weeks of gestation. They also conducted two postpartum home visits: one within the first week after delivery (1–6 days) and another at 6–8 weeks (42–60 days). Women and their children who survived were followed up at different intervals until the children reached 24 months of age.

During the visits, CHWs collected data on women’s demographic and household characteristics, including age, education, husband’s education, household size, and socioeconomic status. Throughout the pregnancy and postpartum visits, CHWs also recorded information on the ongoing pregnancy, maternal weight and height, pregnancy outcome, and conducted depression screening using the Patient Health Questionnaire – 9 (PHQ-9), a validated, widely-used tool in Bangladesh [16].

Two female assessors experienced in developmental assessments conducted child developmental assessments at 24 months of age using the Bayley Scales of Infant and Toddler Development, Third Edition (Bayley III) [17]. The assessors were university graduates in social sciences and had underwent hands-on training in using the Bayley III and attended supervised field practice for three weeks under the supervision of a trained psychologist before the start of data collection. Inter-rater reliability between the assessors and the trainer, evaluated in 20 non-study children, was high (intra-class correlation coefficient >0.98 for both). Assessments were conducted in a controlled field-office environment (quiet and well-ventilated room) with mothers present alongside their children. For quality control, approximately 8–10% of assessments were directly observed and independently scored by the supervisors certified by the WHO in performing the Bayley III assessment throughout the study period. Refresher training was conducted every six months, or earlier if assessor–trainer agreement fell below 90%.

We converted the Bayley III raw scores first into scaled scores and then into composite scores, according to the Bayley III conversion guidelines [18]. The Bayley III language and motor composite scores (range = 45–155) are derived from sums of the subtest scale scores, while the cognitive composite score (range = 55–145) is generated from a single scale score. We retained composite scores for each domain of development as a continuous variable for our analysis.

### Measurements

The primary outcome variables were children's developmental outcomes at 24 months of age, assessed using the Bayley III. Development was evaluated across three domains: cognitive, language, and motor development, using the corresponding composite scores.

The main explanatory variable was depressive symptoms in women, assessed using the PHQ-9 scale at 42–60 days and at 12 months postpartum. The PHQ-9 comprises nine questions that measure the frequency of depressive symptoms over the past two weeks. Responses to each question range from 0 (not at all) to 3 (nearly every day), yielding a total score between 0 and 27, where scores 0–4 indicate none to minimal depression, 5–9 indicate mild depression, 10–14 indicate moderate depression, 15–19 indicate moderately severe depression, and 20–27 indicate severe depression [16]. For this analysis, we recategorised these score groups as follows: no depressive symptoms (score 0–4 at both 6 weeks and 12 months), depressive symptoms (score ≥5 at either 6 weeks or 12 months), and persistent depressive symptoms (score ≥5 at both time points).

#### Other covariates

We categorised maternal age into 15–19 years, 20-29 years, and 30–49 years; maternal education as none, primary education (grades 1–5), and secondary education and above (grade 6 or above); and parity as having 1, 2–3 children, and ≥4 children. We calculated the maternal body mass index (BMI) by dividing weight in kilograms by height in meters squared (kg/m^2^), after which we classified women as underweight (<18.5 kg/m^2^), normal weight (18.5–24.9 kg/m^2^), and overweight or obese (≥25.0 kg/m^2^). Lastly, we generated a household wealth index through principal component analysis based on data on household demographics, parental occupation, education, drinking water, toilet facilities, and assets, after which we used the final household wealth indices to classify households into wealth quintiles.

### Data analysis

We treated the primary exposure variable as a categorical variable and the outcomes (Bayley III scores) as continuous variables. We compared the participants’ maternal and household characteristics (maternal age, education, BMI, and parity; paternal education and household wealth) by the persistence of depressive symptoms in women using chi-squared tests. Specifically, for the chi-squared tests, we tabulated the mean composite scores in each domain (cognitive, language, motor) at 24 months by levels of maternal depressive symptoms (PHQ-9 scores) at different postnatal visits.

Using linear regression models, we assessed the association between the number of times women had depressive symptoms (PPDS) and child development outcomes. We conducted multivariable regression analyses, adjusting for covariates identified *a priori* (maternal age, education, BMI, and preterm birth) and estimating adjusted coefficient values with 95% confidence intervals (CIs). We assessed cognitive, language, and motor outcomes using standardised composite scores from the Bayley III (mean (x̄) = 100, SD = 15); the regression coefficients therefore represent mean differences in these standardised scores between exposure groups. We performed all statistical analyses in Stata, version 17 (StataCorp LLC, College Station, Texas, USA).

## RESULTS

**Figure 1:**
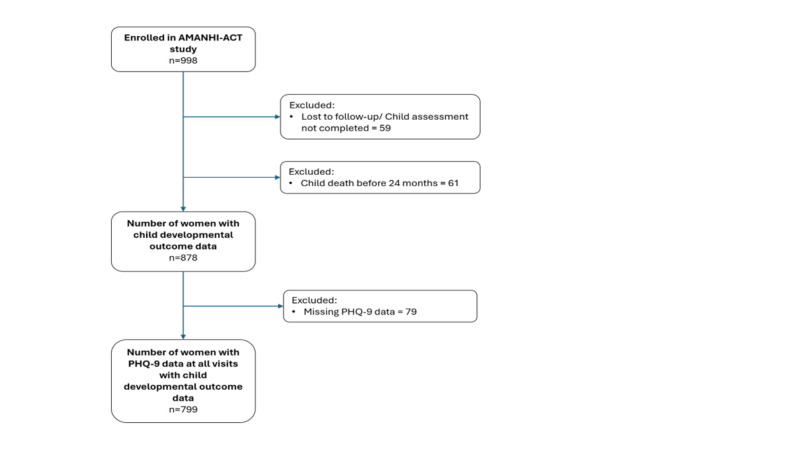
Study flowchart.

The analysis included 998 pregnant women from Sylhet, Bangladesh, who were followed for 24 months. We excluded women whose children died (n = 61), who were lost to follow-up at 24 months (n = 59), and did not have their PHQ-9 measured at any of the postnatal visits at 42–60 days, at 12 months, or 24 months after delivery (n = 79), leaving 799 participants in the final analysis (**Figure 1**). Most of these women reported having depressive symptoms, with 47% and 40% reporting depressive symptoms in one and both visits, respectively. Maternal education and history of any tobacco use, as well as the household wealth index, were found to be significantly associated with depression in the women at one or both visits (**Table 1**).

**Table 1:** Demographic characteristics of women by depressive symptoms assessed between 42-60 days and 12 months postpartum, n (%)

	No depressive symptoms (n = 104)	Depressive symptoms at one visit (n = 375)	Depressive symptoms at both visits (n = 320)	*P*-value
**Maternal age in years**				0.33
15–19	14 (13.5)	79 (21.1)	66 (20.6)	
20–29	75 (72.1)	249 (66.4)	222 (69.4)	
30–49	15 (14.4)	47 (12.5)	32 (10.0)	
**Maternal education**				0.07
None	0 (0.0)	13 (3.5)	18 (5.6)	
Primary (1–5 years)	31 (29.8)	132 (35.2)	110 (34.4)	
Secondary and above (≥6 years)	73 (70.2)	230 (61.3)	192 (60.0)	
**Maternal BMI**				0.10
Underweight (<18.5 kg/m^2^)	19 (18.3)	118 (31.5)	104 (32.5)	
Normal weight (18.5–24.9 kg/m^2^)	76 (73.1)	236 (62.9)	195 (60.9)	
Overweight (25.0–29.9 kg/m^2^)	9 (8.7)	19 (5.1)	18 (5.6)	
Obesity (≥30 kg/m^2^)	0 (0.0)	2 (0.5)	3 (0.9)	
**Parity category**				1.00
0 (primiparous)	36 (34.6)	136 (36.3)	120 (37.5)	
1	30 (28.8)	104 (27.7)	83 (25.9)	
2–3	29 (27.9)	105 (28.0)	92 (28.7)	
≥4	9 (8.7)	30 (8.0)	25 (7.8)	
**Child’s sex**				0.55
Male	47 (45.2)	178 (47.5)	162 (50.6)	
Female	57 (54.8)	197 (52.5)	158 (49.4)	
**Any tobacco use**				0.04
Yes	9 (8.7)	71 (18.9)	58 (18.1)	
No	95 (91.3)	304 (81.1)	262 (81.9)	
**Preterm delivery**				0.20
No	96 (92.3)	329 (87.7)	292 (91.2)	
Yes	8 (7.7)	46 (12.3)	28 (8.8)	
**Paternal education **				0.51
None	12 (11.5)	49 (13.1)	54 (16.9)	
Primary (1–5 years)	52 (50.0)	197 (52.5)	160 (50.0)	
Secondary and above (>5 years)	40 (38.5)	129 (34.4)	106 (33.1)	
**Wealth index**				0.05
Lowest	22 (21.2)	65 (17.3)	71 (22.2)	
Lower	14 (13.5)	81 (21.6)	65 (20.3)	
Middle	20 (19.2)	73 (19.5)	68 (21.2)	
Higher	15 (14.4)	78 (20.8)	62 (19.4)	
Highest	33 (31.7)	78 (20.8)	54 (16.9)	

BMI – body mass index

Children of women who reported no/minimal depressive symptoms during the second visit showed higher cognitive (x̄ = 89.15), language (x̄ = 92.62), and motor scores (x̄ = 92.88) than the children of women who exhibited mild, moderate, or severe depressive symptoms. For language composite scores, we observed a trend of decreasing scores with higher depression categories. We observed similar higher values at the 12-month visit in children of women who reported no depression (Table S1 in the **Online Supplementary Document**).

Children of women who reported no depressive symptoms consistently had higher standardised scores across cognitive, language, and motor domains, with the lowest scores found in the children of women who had depressive symptoms at both visits (cognitive: x̄ = 88.03; language: x̄ = 90.82; motor domain: x̄ = 92.70). Cognitive and language scores showed a trend toward lower scores in women with persistent PPDS (**Table 2**).

**Table 2:** Child development outcomes by different domains at 24 months according to PHQ-9 categories*

	Total (n = 799), n (%)	Cognitive composite score	Language composite score	Motor composite score
**PHQ-9 score-categories at second visit (42–60 d)**				
None/minimal (0–4)	199 (24.91)	89.15 (7.11)	92.62 (8.38)	92.88 (6.87)
Mild (5–9)	520 (65.08)	88.32 (7.01)	90.90 (9.05)	93.15 (6.69)
Moderate to severe (10+)	8 (10.01)	88.75 (6.19)	90.85 (7.60)	93.13 (6.14)
**PHQ-9 score-categories at 12-mo visit**				
None/minimal (0–4)	384 (48.06)	89.19 (6.58)	91.91 (8.85)	93.54 (6.86)
Mild (5–9)	341 42.68)	87.86 (7.52)	90.84 (8.92)	92.52 (6.56)
Moderate to severe (10+)	74 (9.26)	88.58 (5.82)	90.46 (7.50)	93.32 (6.12)

PHQ-9 – Patient Health Questionnaire – 9, SD – standard deviation, x̄ – mean

*Presented as mean (standard deviation) unless specified otherwise.

In the adjusted regression model, the children of women who experienced depressive symptoms at one visit had a statistically significant lower cognitive score (regression coefficient (*β*) = −1.54; 95% CI = −3.05, −0.03), while the children of women with depressive symptoms at both visits had about two points lower score (−2.06; 95% CI = −3.60, −0.52) compared to children of women who experienced no depression. The language scores for children of women with depressive symptoms at one visit were also significantly lower (*β* = −3.01; 95% CI = −4.85, −1.17). This effect was similar among children of mothers with depressive symptoms at both visits (*β* = −3.13; 95% CI = −5.01, −1.25). We found no statistically significant difference for the motor (**Table 3**).

**Table 3:** Effect of persistent postpartum depressive symptoms (at two survey rounds) using PHQ-9 score ≥5 on child development outcomes,

		Unadjusted regression coefficient (95% CI)			Adjusted regression coefficient (95% CI)*		
**Depressive symptoms**	**Total (n = 799), n (%)**	**Cognitive composite score**	**Language composite score**	**Motor composite score**	**Cognitive composite score**	**Language composite score**	**Motor composite score**
No	104 (13.02)	ref	ref	ref	ref	ref	ref
At one visit	375 (46.93)	−1.80 (−3.31, −0.30)	−3.56 (−5.45, −1.67)	0.16(−1.29, 1.61)	−1.54 (−3.05, −0.03)	−3.01 (−4.85, −1.17)	0.34 (−1.11, 1.80)
At two visits	320 (40.05)	−2.31 (−3.84, −0.77)	−3.63 (−5.55, −1.70)	−0.51 (−1.99, 0.97)	−2.06 (−3.60, −0.52)	−3.13 (−5.01, −1.25)	−0.37 (−1.86, 1.12)

CI – confidence interval, PHQ-9 – Patient Health Questionnaire – 9, ref – reference

*Adjusted for maternal age, education, BMI, and preterm birth

## DISCUSSION

We examined how PPDS affected children’s cognitive, language, and motor development at two years of age in a cohort of women from Sylhet, Bangladesh, and found significant negative effects of PPDS on children’s cognitive and language scores.

The literature on postpartum depression and child development is conflicting, with some studies reporting an effect [11,12,19–21] and others not [22,23]. Most studies on PPDS and child development were conducted in high-income countries [12], with limited data from low- and middle-income countries (LMICs) [19]. A study by Netsi *et al*. [12] in the UK observed that chronic maternal depression was associated with increased behavioural issues (odds ratio (OR) = 4.84; 95% CI = 2.94, 7.98). Sutter-Dallay *et al*. [19] reported that PPDS among women in France negatively impacted children’s cognitive development by age two, which is similar to what we found in this study. Quevedo *et al*. [11] in Brazil and Abdollahi *et al*. [20] in Iran similarly observed that chronic postpartum depression led to poor language development (OR = −2.87; 95% CI = −5.01, −0.64), as well as lower gross motor domain (OR = 4.15; 95% CI = 2.72, 13.87) and personal-social domain (OR = 6.17; 95% CI = 1.95, 19.53) scores from 12 months to 4 years. In Brazil, Santos *et al*. [21] reported results similar to ours, with a decrease in Ages and Stages Questionnaire scores among children with chronicity of depression at three years during follow-up.

In contrast, other studies reported no significant effect of PPDS on child development outcomes. While Nasreen *et al*. [22] found that maternal depression in early infancy was linked to delayed motor development among children in rural Bangladesh, they observed no effect with persistent depression at 6–8 months (β = −498; standard error = 0.255, *P* = 0.052) . Kurstjens *et al*. [23] studied 1329 mothers in Germany and also found negligible effects of chronic maternal depression on children’s cognitive development, with effects only seen if the child was born in lower socioeconomic status families. The differences in findings between these studies and ours could be attributed to variation in population characteristics, timing of assessment, tools used, and specific developmental domains examined.

Our study contributes to the evidence documenting that PPDS has a negative impact on child development in LMICs, which may stem from the compounding effect of poverty, malnutrition, and limited access to healthcare services. As such, it has several strengths. The cohort design and longitudinal follow-up suggest that there is a temporal relationship between PPDS and child development. The PHQ-9 and Bayley III have both been validated and widely used in Bangladesh [24,25]. However, we also acknowledge some limitations. First, maternal PPDS symptoms were assessed based on self-reporting, which could be subject to reporting bias. Although the PHQ-9 is a validated instrument in this context, future studies could benefit from triangulation with clinical assessments to enhance measurement accuracy. Second, while we adjusted for a range of demographic and socioeconomic factors, residual confounding may remain from unmeasured variables as cultural influences, paternal or broader caregiving characteristics, and breastfeeding practices. Third, we conducted this study within a specific rural setting in Bangladesh; results may vary in different LMIC contexts, so the generalisability of our findings may be limited. Fourth, while excluded participants were largely similar to the analytic sample, preterm birth was more common among those excluded, suggesting a potential selection bias. Additionally, we assessed maternal depressive symptoms at only two postpartum time points (at 6 weeks and 12 months), with PPDS consequently defined as the presence of symptoms at both assessments. This limited measurement frequency may not fully capture changes in depressive symptoms over time and may have resulted in some misclassification between episodic and persistent depression. Further research is needed to explore the mechanisms linking PPDS to child developmental outcomes, particularly in LMICs. Studies examining the role of caregiving environments, stimulation, and cultural practices could also provide valuable insights, as would extending follow-up beyond 24 months to assess long-term developmental trajectories.

Evidence from Bangladesh demonstrates that maternal depression is associated with delayed motor development and adverse child health outcomes, highlighting the urgency of local interventions [22]. Maternal depression in LMICs is often exacerbated by socioeconomic challenges, malnutrition, and limited access to healthcare services [26]. Addressing PPDS not only improves maternal well-being, but also fosters optimal child development, potentially breaking intergenerational cycles of poverty and poor health outcomes [27]. Interventions such as community-based mental health programmes, early identification, and treatment of depressive symptoms, combined with promoting stimulating caregiving environments, are essential for mitigating these adverse effects [28,29]. Furthermore, integrating maternal mental healthcare into primary health systems aligns with global priorities for improving child developmental outcomes [2]. Scaling up such interventions could yield significant benefits for achieving global developmental goals, reducing health inequities, and improving the future potential of children in LMICs. Our findings highlight the public health importance of addressing persistent PPDS in LMICs like Bangladesh. Maternal mental health is a key determinant of early childhood development, with persistent PPDS negatively affecting cognitive and language outcomes in children. This underscores the need for integrating mental health screening into maternal and child health programs, particularly in resource-limited settings. Bangladesh’s National Mental Health Strategic Plan (2020–2030) [30] emphasises integrating mental health into primary care through task-sharing, whereby brief, validated tools such as the PHQ-9 and Generalized Anxiety Disorder – 7 are used by CHWs during routine antenatal and postnatal visits.

## CONCLUSIONS

In this rural Bangladeshi cohort, PPDS was associated with adverse child developmental outcomes. These findings highlight the importance of addressing maternal mental health to support optimal early childhood development.

## Additional material


Online Supplementary Document
