# Supplementary material for: Persistent postpartum depressive symptoms and developmental impairment in Bangladeshi children: a cohort study
Source: J Glob Health. 2026 Jul 3;16:04207. doi: 10.7189/jogh.16.04207 (PMC13329880; doi:10.7189/jogh.16.04207)
Supplement: Online Supplementary Document [file jogh-16-04207-s001.pdf]

Supplement to: Khanam R, Lazar VMA, Chowdhury NH, Ahmed S, Tofail F, Khan RA, Surkan PJ, Mohan D, Yoshida S, Sazawal S, Zehan F, Baqui AH. Persistent postpartum depressive symptoms and developmental impairment in Bangladeshi children: a cohort study. J Global Health. 2026;16:04207.

Table S1. Cognitive, language, and motor composite scores of children at 24 months by persistent postpartum depressive symptoms*

Depressive symptoms	Total (n = 799), n (%)	Cognitive composite score	Language composite score	Motor composite score
No	104 (13.02)	90.34 (6.98)	94.44 (8.91)	93.21 (7.94)
At one visit	375 (46.93)	88.53 (6.57)	90.89 (8.32)	93.37 (6.22)
At two visits	320 (40.05)	88.03 (7.31)	90.82 (9.06)	92.70 (6.76)

SD – standard deviation, $\bar{x}$ – mean

*Presented as mean (standard deviation) unless specified otherwise.
